# Association of Plasma IL-6 and Hsp70 with HRV at Different Levels of PAHs Metabolites

**DOI:** 10.1371/journal.pone.0092964

**Published:** 2014-04-10

**Authors:** Jian Ye, Rui Zhu, Xiaosheng He, Yingying Feng, Liangle Yang, Xiaoyan Zhu, Qifei Deng, Tangchun Wu, Xiaomin Zhang

**Affiliations:** 1 Department of Occupational and Environmental Health and Ministry of Education Key Lab for Environment and Health, School of Public Health, Tongji Medical College, Huazhong University of Science and Technology, Wuhan, China; 2 Department of Traditional Chinese Medicine, Union Hospital, Tongji Medical College, Huazhong University of Science and Technology, Wuhan, China; Harvard Medical School, United States of America

## Abstract

**Background:**

Exposure to polycyclic aromatic hydrocarbons (PAHs) is associated with reduced heart rate variability (HRV), a strong predictor of cardiovascular diseases, but the mechanism is not well understood.

**Objectives:**

We hypothesized that PAHs might induce systemic inflammation and stress response, contributing to altered cardiac autonomic function.

**Methods:**

HRV indices were measured using a 3-channel digital Holter monitor in 800 coke oven workers. Plasma levels of interleukin-6 **(**IL-6) and heat shock protein 70 (Hsp70) were determined using ELISA. Twelve urinary PAHs metabolites (OH-PAHs) were measured by gas chromatography-mass spectrometry.

**Results:**

We found that significant dose-dependent relationships between four urinary OH-PAHs and IL-6 (all *P*_trend_<0.05); and an increase in quartiles of IL-6 was significantly associated with a decrease in total power (TP) and low frequency (LF) (*P*_trend_ = 0.014 and 0.006, respectively). In particular, elevated IL-6 was associated in a dose-dependent manner with decreased TP and LF in the high-PAHs metabolites groups (all *P*_trend_<0.05), but not in the low-PAHs metabolites groups. No significant association between Hsp70 and HRV in total population was found after multivariate adjustment. However, increased Hsp70 was significantly associated with elevated standard deviation of NN intervals (SDNN), TP and LF in the low-PAHs metabolites groups (all *P*_trend_<0.05). We also observed that both IL-6 and Hsp70 significantly interacted with multiple PAHs metabolites in relation to HRV.

**Conclusions:**

In coke oven workers, increased IL-6 was associated with a dose-response decreased HRV in the high-PAHs metabolites groups, whereas increase of Hsp70 can result in significant dose-related increase in HRV in the low-PAHs metabolites groups.

## Introduction

Polycyclic aromatic hydrocarbons (PAHs) are the major component of coke oven emissions produced during incomplete combustion of natural or synthetic fuels [1], [2]. Recent studies, including those from our lab [3], [4], found that increased exposure to PAHs was associated with reduced cardiac autonomic function, assessed by heart rate variability (HRV), which is considered one of the main pathophysiologic pathways for air pollution-mediated adverse cardiac events [5]–[8]. However, the mechanisms through which the cardiac autonomic system responds to PAHs exposure have not been sufficiently understood.

Systemic inflammation may be one of the potential mechanisms linking air pollution including PAHs to cardiovascular autonomic decline. It has been reported that elevated levels of inflammation biomarkers, especially that of C-reactive protein (CRP) and interleukin-6 (IL-6), are inversely associated with various HRV indices in apparently normal adults [9]–[11] and in patients with coronary heart disease (CHD) [12], [13]. However, to our knowledge, no studies have evaluated the relationship between inflammation and HRV in occupational population exposed to different levels of PAHs.

Heat shock protein 70 (Hsp70), one of the main HSP family members, has a dual role as chaperone and cytokine by inducing pro-inflammatory cytokines such as IL-6 production [14]. A number of studies have shown that circulating Hsp70 play a crucial role in the pathogenesis and/or prognosis of cardiovascular diseases [15]–[18]. We previously found that exposure to PAHs, as reflected by urinary 1-hydroxypyrene (1-OHP), resulted in a dose-dependent increase in levels of plasma Hsp70, suggesting that high Hsp70 levels may serve as a danger marker among workers exposure to PAHs [19]. We thus hypothesized that circulating Hsp70 may be associated with cardiac autonomic function in response to occupational PAHs exposure.

To test the above hypothesis, we examined the association of urinary monohydroxylated PAHs (OH-PAHs) with plasma IL-6 and Hsp70, and the effects of IL-6 and Hsp70 on HRV in 800 workers exposed to different levels of PAHs.

## Materials and Methods

### Ethics Statement

The study was approved by the Ethics and Human Subject Committee of Tongji Medical College and informed written consent was obtained from each subject.

### Study Subjects

The cross-sectional survey was conducted throughout the workday (Monday to Friday) from Oct to Nov 2009 in Wuhan (Hubei, China). Eight hundred healthy workers who had worked for at least one year at different sites, including at the top, side, bottom, or adjacent workplaces of coke ovens, and at the offices, in the same coke oven plant, were included in this study. Subjects who had suffered from cardiovascular diseases, serious medical conditions affecting HRV or chronic inflammation in the previous 3 months were excluded from the study. In the morning, workers visited the occupational health examination center. 5 mL of venous blood and 20 mL of urine samples were collected at the beginning (pre-shift) of workshift from the workers who were on the day shift, and the end (post-shift) of workshift from those who were on the night shift. All blood and urine samples were divided into aliquots and frozen at −80°C until laboratory analysis. Questionnaires, physical examination and Holter monitoring followed to be completed. Information on demographic variables, occupational history, medications, history of disease, and lifestyle habits including smoking, passive smoking, alcohol consumption, physical activity, and diet were collected using standardized occupational questionnaires.

### Determination of Urinary PAHs Metabolites

We measured 12 urinary PAHs metabolites [pyrene metabolite: 1-hydroxypyrene (1-OHP); naphthalene metabolites: 1-hydroxynaphthalene (1-OHNa), 2-OHNa; fluorene metabolites: 2-hydroxyfluorene (2-OHFlu), 9-OHFlu; phenanthrene metabolites: 1-hydroxyphenanthrene (1-OHPh), 2-OHPh, 3-OHPh, 4-OHPh, 9-OHPh; chrysene metabolite: 6-hydroxychrysene (6-OHChr); and benzo[a]pyrene metabolite: 3-hydroxybenzo[a]pyrene (3-OHBaP)] by gas chromatography-mass spectrometry (GC/MS, Agilent 6890N+5975B, Agilent Technologies Inc., Santa Clara, CA, USA) as previously described [4]. Briefly, each 3.0 mL urine sample was extracted three times to elevate the detection rate. The set of the standard curve was operated about every 100 samples and re-run about 10% of the total samples were used as quality control. The identification and quantification of urinary PAHs metabolites were based on retention time, mass-to-charge ratio, and peak area using a linear regression curve obtained from separate internal standard solutions. The limits of detection (LOD) for the urinary PAHs metabolites were in the range 0.1–1.4 μg/L; default values were replaced with 50% of the LOD. Valid urinary PAHs metabolite concentrations were calibrated by levels of urinary creatinine and calculated as μg/mmol creatinine. Because 6-OHChr and 3-OHBaP were below the limits of quantification, we only analyzed 10 metabolites of PAHs.

### Detection of IL-6 and Hsp70 in Plasma

IL-6 and Hsp70 levels were measured in EDTA-plasma using IL-6 and Hsp70 enzyme-linked immunosorbent assay (ELISA) kits (NeoBioscience Technology Company, China and Stressgen Bioreagents Company, Victoria, BC, Canada). The Hsp70 assay only detects inducible Hsp70 and does not detect other Hsp70 family members such as constitutive Hsp70, Grp78, DnaK (*Escherichia coli*), or Hsp71 (*Mycobacterium tuberculosis*). The inter-assay and intra-assay coefficients of variation of the assays were <10%.

### Measurements of HRV

The measurement of HRV was conducted between 8∶00 am and 11∶00 am and measurement methods have been described previously [4]. After at least 5-min rest, each participant was seated comfortably on a chair and was fitted with a 3-channel digital Holter monitor (Lifecard CF; Del Mar Reynolds Medical, Inc., Irvine, USA) with a 1024 samples/second sampling rate for 10 minutes. We cleaned the participant’s skin with an alcohol wipe and abraded slightly to keep good lead contacts, and placed the separate electrode in the position according to the instruction and technical manual of Lifecard CF. All of the HRV indices were calculated on 5-min epoch in the entire recording. Only heart rates between 40 and 100 beats per minute were submitted to analyses [20]. We selected five consecutive minutes of ECG reading in the statistical analysis without atria and ventricular premature beats and flutter. The HRV spectrum was computed with a fast Fourier transform method and analyzed in both time and frequency domains. The measured time domain parameters include: a) SDNN (standard deviation of NN intervals in milliseconds), is an estimation of total HRV power; b) RMSSD (the root mean of square of successive differences between adjacent normal NN intervals in milliseconds), reflects the activities of parasympathetic nervous system. The frequency-domain variables include: a) low-frequency (LF msec^2^; 0.04–0.15Hz) may represent the combination of both parasympathetic and sympathetic activity of heart rate; b) high-frequency (HF msec^2^; 0.15–0.4Hz) shows the actions of the parasympathetic modulation of heart rate; c) total power (TP msec^2^; approximately≤0.4Hz) is a mixture of total variability of heart rate.

### Statistical Analyses

We assessed the normality of all variables with the one-sample K-S test. Normal distributions of HRV indices and values of all urinary PAHs metabolites and plasma IL-6 and Hsp70 were obtained by natural logarithmic transformation. Because there is no well-established cutoff point for defining high versus low total OH-PAHs exposure, while 33^rd^ and 67^th^ percentiles [1] or the interquartile range (75^th^ vs. 25^th^ percentile) [21] was used as cutoffs in previous studies, we dichotomized total OH-PAHs at the 33th percentile as either low or high. Plasma IL-6 and Hsp70 were considered as categorical variables at different cut-off points. Baseline characteristics between urinary PAHs metabolites categories were compared using *t*-tests for continuous variables and chi-square tests for categorical variables. The trend for urinary PAHs metabolites levels in the four environmental exposure groups were tested by using simple linear regression. Pearson partial correlation coefficients (r) were computed to examine the associations among ln-transformed individual PAHs metabolites and ΣOH-PAHs. The trend for IL-6 and Hsp70 among quartiles of PAHs metabolites and the trend for HRV among quartiles of the IL-6 and Hsp70 were tested by multivariate linear regression models. Moreover, effects of IL-6 and Hsp70 on HRV stratified by PAHs metabolites were estimated using general linear models. Finally, to analyze potential interactions between IL-6 and PAHs metabolites as well as Hsp70 and PAHs metabolites, interaction product terms were included in the model. Potential confounders, including age, sex, length of work, smoking status, alcohol use, BMI, physical activity, working sites (top-oven, side and bottom-oven, adjunct-oven, and at the offices), and workshift (pre-shift and post-shift) for acute exposure – over the course of a day and weekday (Monday to Friday) for intermediate-term exposure – over the course of a week were used in the analysis. All statistical tests were derived from two-sided analyses and *P*<0.05 was considered as statistical significance. All analyses were conducted using SPSS (version 12.0).

## Results

### Characteristics of Study Population in Relation to Urinary Total OH-PAHs Levels

General characteristics of the workers classified by urinary total OH-PAHs levels are presented in Table 1. The distribution according to age, length of work, current smoker, current alcohol drinker, BMI and physical activity in the two groups was similar (all *P*>0.05), but differed in sex and workshift (pre-shift) (*P*<0.001). The median level of IL-6 was lower in the low total OH-PAHs group compared with the high total OH-PAHs group (1.86 pg/ml vs. 2.22 pg/ml), whereas Hsp70 level was similar. Averages of the five HRV indices were lower among those with the high total OH-PAHs levels, although SDNN and TP were not statistically significant.

**Table 1:** General characteristics of the workers by urinary total OH-PAHs levels*.

Characteristics	ΣOH-PAHs (μg/mmol creatinine)^a^		*P-*value^b^
	Low (n = 266)	High (n = 534)	
Age (years)	40.70±7.77	41.48±8.13	0.199
Sex (male)	243 (91.4)	431 (80.7)	**<0.001**
Length of work (years)	19.91±8.97	20.39±9.58	0.493
Current smoker	150 (56.4)	291 (54.6)	0.631
Current alcohol drinker	84 (31.6)	187 (35.1)	0.324
BMI (kg/m^2^)	23.87±3.78	23.50±3.62	0.174
Physical activity (yes)	131 (50.4)	228 (43.1)	0.053
Workshift (pre-shift)	139 (52.3)	208 (39.0)	**<0.001**
IL-6 (pg/mL)	1.86 (1.20, 3.11)	2.22 (1.29, 3.96)	**<0.001**
Hsp70 (ng/mL)	0.82 (0.16, 1.96)	1.01 (0.32, 1.87)	0.660
HRV indices [mean (SE)]^c^			
SDNN (msec)	3.72 (0.02)	3.69 (0.01)	0.246
RMSSD (msec)	3.23 (0.02)	3.17 (0.01)	**0.027**
TP (msec^2^)	7.09 (0.04)	6.99 (0.03)	0.067
LF (msec^2^)	6.01 (0.05)	5.84 (0.04)	**0.011**
HF (msec^2^)	5.14 (0.06)	4.90 (0.04)	**<0.001**

BMI, body mass index; HF, high frequency; HRV, heart rate variability; Hsp70, heat shock protein 70; IL-6, interleukin-6; LF, low frequency; RMSSD, root mean square of successive differences in adjacent NN intervals; SDNN, standard deviation of NN intervals; TP, total power.

*Values are presented as mean ± SD, n (%), or median (25th, 75th quartile) unless otherwise indicated.

a ΣOH-PAHs was dichotomized at the 33th percentile as either low (≤8.84 μg/mmol creatinine) or high (>8.84 μg/mmol creatinine). ^b^ *P-*values were calculated by χ^2^ test for categorical variables, and t-test for continuous variables. ^c^ HRV indices were natural log transformed.

### Urinary Levels of PAHs Metabolites at Different Working Sites

As our previous report [4], we defined office workers as the control group, and workers at adjunct-oven, and at side and bottom-oven, and top-oven as low, intermediate and high exposure groups, respectively. The concentrations of ΣOH-PAHs and nine urinary PAHs metabolites with the exception of 4-OHPh were significantly elevated with increasing environmental PAHs exposure levels (Table S1 in File S1, all *P*_trend_<0.01).

### Correlations of Urinary PAHs Metabolites

Almost all individual PAHs metabolites were significantly correlated with each other, except 3-OHPh and 9-OHFlu as well as 2-OHPh, 3-OHPh and 4-OHPh. The correlation coefficients between individual PAHs metabolites and ΣOH-PAHs ranged from 0.262 to 0.851. The strongest correlations between individual PAHs metabolites and ΣOH-PAHs was 1-OHNa (r = 0.851), followed by 1-OHP and 2-OHNa (r = 0.836 and 0.772), the lowest correlations was 4-OHPh (r = 0.262) (Table S2 in File S1).

### Effects of PAHs Metabolites on IL-6 and Hsp70 as Well as HRV

**Figure 1:**
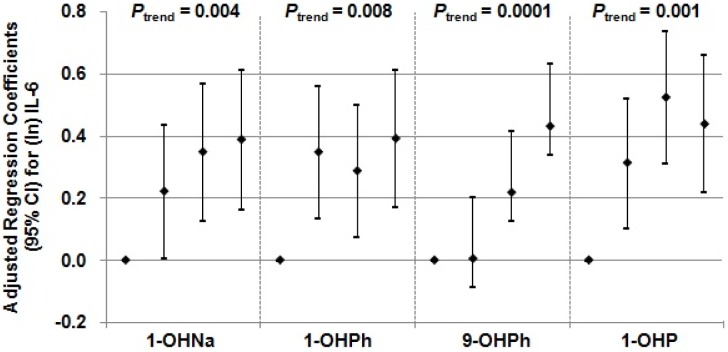
Association of urinary PAHs metabolites quartiles and ln-transformed IL-6. *P*_trend_ was calculated from the regression models, adjusting for age, sex, length of work, smoking status, alcohol use, BMI, physical activity, working sites, workshift and weekday. 1-OHNa: Q1 (≤0.92 μg/mmol creatinine), Q2 (0.92–1.57 μg/mmol creatinine), Q3 (1.57–2.80 μg/mmol creatinine), Q4 (>2.80 μg/mmol creatinine); 1-OHPh: Q1 (≤0.40 μg/mmol creatinine), Q2 (0.40–0.84 μg/mmol creatinine), Q3 (0.84–1.55 μg/mmol creatinine), Q4 (>1.55 μg/mmol creatinine); 9-OHPh: Q1 (≤0.39 μg/mmol creatinine), Q2 (0.39–0.70 μg/mmol creatinine), Q3 (0.70–1.35 μg/mmol creatinine), Q4 (>1.35 μg/mmol creatinine); 1-OHP: Q1 (≤1.86 μg/mmol creatinine), Q2 (1.86–3.29 μg/mmol creatinine), Q3 (3.29–6.11 μg/mmol creatinine), Q4 (>6.11 μg/mmol creatinine).

After controlling for potential confounders included age, sex, length of work, smoking status, alcohol use, BMI, physical activity, working sites, workshift and weekday, increasing quartiles of four urinary OH-PAHs (1-OHNa, 1-OHPh, 9-OHPh and 1-OHP) were significantly associated, in a dose-responsive manner, with increased IL-6 (*P*_trend_ = 0.004, 0.008, 0.0001 and 0.001, respectively) (Figure 1). However, no significant dose-response relationship between PAHs metabolites and Hsp70 was found after multivariate adjustment.

In addition, increasing quartiles of 2-OHNa and 1-OHPh were significantly associated with decreased HF (*P*_trend_ = 0.028 and 0.012, respectively), but no significant association between other metabolites and HRV indices was observed (Table S3 in File S1).

### Dose-dependent Decrease in HRV Associated with Increased IL-6

**Figure 2:**
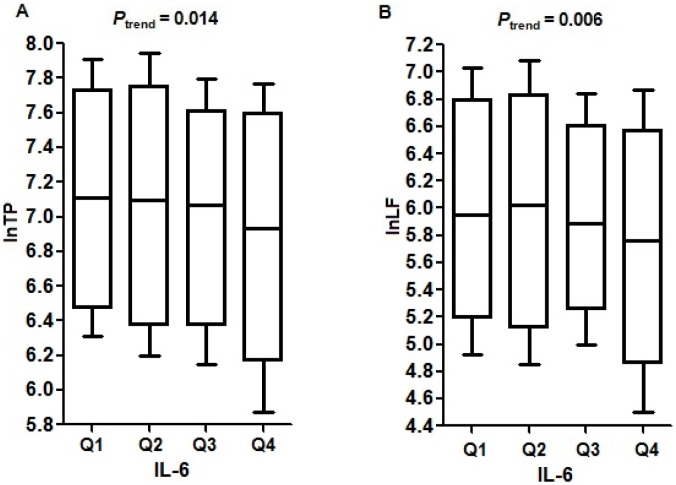
Values for natural log transformed TP (A) and LF (B) by quartiles of IL-6. *P*_trend_ was calculated from the regression models, adjusting for age, sex, length of work, smoking status, alcohol use, BMI, physical activity, working sites, workshift and weekday. Box plots indicate the 10th quartile, 25th quartile, median, 75th quartile, and 90th quartile for each group. IL-6: Q1 (≤1.25 pg/ml), Q2 (1.25–2.13 pg/ml), Q3 (2.13–3.71 pg/ml), Q4 (>3.71 pg/ml).

As shown in Figure 2, after adjustment for covariates, increasing quartiles of IL-6 levels were significantly associated with decreases in TP and LF (*P*_trend_ = 0.014 and 0.006). Compared to the lowest quartile, individuals with the highest quartile of IL-6 were significantly associated with 2.8% and 4.8% reduction in TP and LF.

### Association of IL-6 with HRV by PAHs Metabolites

Given that HRV had significant association with IL-6 levels, we next examined whether the relationship of IL-6 with HRV differed in low- versus high-PAHs metabolites. As shown in Table 2, after adjustment for confounders, we found that elevated IL-6 levels were significantly associated with decreases in TP and LF in the high-PAHs metabolites groups (ΣOH-PAHs, 1-OHP and 2-OHPh, all *P*_trend_<0.05), but not in the low-PAHs metabolites groups. Significant interactions between IL-6 and ΣOH-PAHs on TP (*P*_interaction_ = 0.017), and between IL-6 and 1-OHP, 2-OHPh and ΣOH-PAHs on LF (*P*_interaction_ = 0.027, 0.044 and 0.001, respectively) were also observed.

**Table 2:** Association of plasma IL-6 with HRV by urinary PAHs metabolites in coke oven workers*.

HRV indices	IL6^a^			*P*_trend_^b^	*P*_interaction_^c^
	Tertile 1	Tertile 2	Tertile 3		
	(n = 267)	(n = 267)	(n = 266)		
TP (msec^2^)					
ΣOH-PAHs^d^					**0.017**
Low (n = 266)	6.90 (0.09)	6.99 (0.09)	7.03 (0.10)	0.219	
High (n = 534)	7.02 (0.07)	6.90 (0.07)	6.84 (0.06)	**0.049**	
LF (msec^2^)					
1-OHP^e^					**0.027**
Low (n = 267)	5.65 (0.10)	5.82 (0.11)	5.73 (0.12)	0.563	
High (n = 533)	5.86 (0.08)	5.73 (0.09)	5.60 (0.08)	**0.040**	
2-OHPh^f^					**0.044**
Low (n = 266)	5.68 (0.10)	5.88 (0.11)	5.74 (0.12)	0.875	
High (n = 534)	5.84 (0.08)	5.72 (0.09)	5.61 (0.08)	**0.029**	
ΣOH-PAHs					**0.001**
Low (n = 266)	5.66 (0.10)	5.92 (0.11)	5.86 (0.12)	0.165	
High (n = 534)	5.87 (0.08)	5.70 (0.08)	5.58 (0.08)	**0.012**	

1-OHP, 1-hydroxypyrene; 2-OHPh, 2-hydroxyphenanthrene; ΣOH-PAHs, total concentration of all PAHs metabolites.

*HRV indices were natural log transformed and presented as multivariate adjusted means and SE.

a IL-6 tertile were defined as less than or equal to 1.52 pg/ml, 1.52 to 2.95 pg/ml, and greater than 2.95 pg/ml, respectively. ^b^Multivariate linear regression for the trend of HRV with IL-6 levels with adjustment for age, sex, length of work, smoking status, alcohol use, BMI, physical activity, working sites, workshift and weekday. ^c^General linear models for the interaction between IL-6 and PAHs metabolites on HRV. ^d^ΣOH-PAHs: Low≤8.84 μg/mmol creatinine, High>8.84 μg/mmol creatinine. ^e^1-OHP: Low≤2.20 μg/mmol creatinine, High>2.20 μg/mmol creatinine. ^f^2-OHPh: Low≤0.21 μg/mmol creatinine, High>0.21 μg/mmol creatinine.

### Association of Hsp70 with HRV by PAHs Metabolites

No significant associations between Hsp70 and HRV were found in total population after adjusting for confounders (data not shown). When we examined the relationship of Hsp70 and HRV stratified by PAHs metabolites, we found that there was a significant increase in SDNN, TP and LF associated with Hsp70 when multiple PAHs metabolites were low (including 1-OHP, 1-OHNa, 2-OHPh, 9-OHPh and ΣOH-PAHs, all *P*_trend_ *<*0.05), but not when they were high. Tests for interaction between Hsp70 and multiple PAHs metabolites were significant on SDNN, TP and LF (all *P*_interaction_<0.05) (Table 3).

**Table 3:** Association of plasma Hsp70 with HRV by urinary PAHs metabolites in coke oven workers*.

HRV indices	Hsp70^a^			*P*_trend_b	*P*_interaction_c
	Tertile 1	Tertile 2	Tertile 3		
	(n = 268)	(n = 266)	(n = 266)		
SDNN (msec)					
1-OHP^d^					**0.003**
Low (n = 267)	3.61 (0.04)	3.65 (0.05)	3.72 (0.04)	**0.006**	
High (n = 533)	3.73 (0.03)	3.64 (0.03)	3.66 (0.03)	0.149	
1-OHNa^e^					**0.002**
Low (n = 266)	3.63 (0.04)	3.68 (0.04)	3.74 (0.04)	**0.005**	
High (n = 534)	3.72 (0.03)	3.64 (0.03)	3.66 (0.03)	0.220	
ΣOH-PAHs^f^					**0.027**
Low (n = 266)	3.65 (0.04)	3.67 (0.04)	3.73 (0.04)	**0.024**	
High (n = 534)	3.71 (0.03)	3.64 (0.03)	3.66 (0.03)	0.433	
TP (msec^2^)					
1-OHP					**0.030**
Low (n = 267)	6.81 (0.08)	6.91 (0.10)	7.01 (0.09)	**0.013**	
High (n = 533)	7.03 (0.07)	6.85 (0.06)	6.94 (0.07)	0.541	
1-OHNa					**0.018**
Low (n = 266)	6.85 (0.09)	6.95 (0.09)	7.08 (0.09)	**0.007**	
High (n = 534)	7.01 (0.07)	6.84 (0.06)	6.93 (0.07)	0.532	
2-OHPh^g^					**0.006**
Low (n = 266)	6.82 (0.08)	6.99 (0.09)	7.07 (0.09)	**0.003**	
High (n = 534)	7.04 (0.07)	6.83 (0.06)	6.93 (0.07)	0.323	
LF (msec^2^)					
1-OHNa					**0.040**
Low (n = 266)	5.71 (0.10)	5.84 (0.11)	5.94 (0.11)	**0.023**	
High (n = 534)	5.78 (0.08)	5.64 (0.08)	5.69 (0.08)	0.450	
2-OHPh					**0.001**
Low (n = 266)	5.58 (0.10)	5.83 (0.12)	5.92 (0.11)	**<0.001**	
High (n = 534)	5.85 (0.08)	5.65 (0.08)	5.69 (0.08)	0.138	
9-OHPh^h^					**0.023**
Low (n = 266)	5.60 (0.10)	5.74 (0.12)	5.84 (0.11)	**0.007**	
High (n = 534)	5.82 (0.08)	5.67 (0.08)	5.71 (0.08)	0.326	

1-OHNa, 1-hydroxynaphthalene; 1-OHP, 1-hydroxypyrene; 2-OHPh, 2-hydroxyphenanthrene; 9-OHPh, 9-hydroxyphenanthrene; ΣOH-PAHs, total concentration of all PAHs metabolites.

*HRV indices were natural log transformed and presented as multivariate adjusted means and SE.

a Hsp70 tertile 1 through 3 were defined as less than or equal to 0.47 ng/ml, 0.47 to 1.52 ng/ml, and greater than 1.52 ng/ml, respectively. ^b^Multivariate linear regression for the trend of HRV with Hsp70 levels with adjustment for age, sex, length of work, smoking status, alcohol use, BMI, physical activity, working sites, workshift and weekday. ^c^General linear models for the interaction between Hsp70 and PAHs metabolites on HRV. ^d^1-OHP: Low≤2.20 μg/mmol creatinine, High>2.20 μg/mmol creatinine. ^e^1-OHNa: Low≤1.09 μg/mmol creatinine, High>1.09 μg/mmol creatinine. ^f^ΣOH-PAHs: Low≤8.84 μg/mmol creatinine, High>8.84 μg/mmol creatinine. ^g^2-OHPh: Low≤0.21 μg/mmol creatinine, High>0.21 μg/mmol creatinine. ^h^9-OHPh: Low≤0.48 μg/mmol creatinine, High>0.48 μg/mmol creatinine.

## Discussion

Increasing epidemiological evidences have indicated significant relationship between inflammatory markers, most commonly CRP and IL-6, and various HRV indices in CHD patients [12], [13], type I diabetes [22], and in apparently normal adults [9], [11].However, until now, the association between inflammatory markers and HRV in workers exposed to different levels of PAHs has been less clear. We hypothesized that inflammation and stress response might play an important role in cardiac autonomic dysfunction induced by PAHs and potential dose-response relationships among them. In the present study, we used IL-6 as a marker to test this hypothesis, because that IL-6 is a good indicator of cytokine cascade activation, accurately reflects the inflammatory status, and is stable as its half-life is longer than other proinflammatory cytokines [23]. We found a significant dose-dependent relationship between four urinary OH-PAHs and IL-6; and IL-6 was significantly associated, in a dose-responsive manner, with decreased HRV, suggesting that certain OH-PAHs rather than others may be a significant contributor to the autonomic dysfunction through an inflammation mechanism. What is new and intriguing about our results is there were significant interactions between some PAHs metabolites and IL-6 on HRV. The link between IL-6 and HRV was only significant in workers with high-PAHs metabolites, but not in those with low-PAHs metabolites, indicating that high-PAHs exposure amplified the association of inflammation and HRV.

PAHs have been documented as one of the most important components of coke oven emission. Measurement of the urinary OH-PAHs metabolites, as internal markers, is an important way of assessing exposure to PAHs, since it takes into account all absorption routes of exogenous compounds [24], [25]. Due to relatively short half-lives of PAHs, the information provided by biomonitoring of urinary OH-PAH is limited to recent exposure [26], [27]. Most potential health effects are likely associated with exposure over time, thus considering the exposure time is essential for data interpretation, especially for chemicals with short half-lives. Li et al [26] evaluated the variability of nine urinary OH-PAHs metabolites over time in non-occupationally exposed subjects and found that the within-day variance exceeded the between-day variance, and the between-subject variance out-weighted the between-day variance for certain metabolites. However, our results did not substantially change even after adjusting for indicator variables of exposure time such as workshift for potential acute exposure (over the course of a day) and weekday intermediate-term exposure (over the course of a week), which strengthened the robustness of the observed association.

Studies have shown that exposure to high levels PAHs was positively associated with the prevalence, mortality or morbidity of cardiovascular disease[1], [28], and one mechanism by which PAHs may play a role in the development of cardiovascular disease is through inflammation [28]–[30]. Thus, high-PAHs exposure has been speculated to contribute to increasing systemic inflammation and subsequent reduced autonomic function, which might explain the significant relationship between IL-6 and HRV in high-PAHs exposure. The hypothesis is supported by studies that have identified that elevated PAHs levels were associated with increased IL-6 or CRP in non-occupational exposure population [29], [31], and systemic inflammatory activity was associated with a decrease in HRV [11], [32]. However, previous studies have only focused on one aspect of the relationships among PAHs, inflammatory biomarkers or HRV; no studies so far have examined all of them together, thus we cannot compare directly with that of other published studies. Luttmann-Gibson et al [33] showed that systemic inflammation modified susceptibility to air pollution-associated autonomic dysfunction in elderly subjects, but the cross-sectional nature of our study precludes any inference about the causal direction of the association between inflammation and HRV related to PAHs exposure. Together with the results of prior studies, these findings make it clear that significant decreased HRV is indeed associated with inflammation among workers with high-PAHs exposure, implicating that pollution can lead to autonomic dysfunction through inflammation.

Intracellular Hsp70 has been found to provide a spectrum of protection against any of a variety of stresses, preventing damage of cells and tissues [34], [35], while plasma extracellular Hsp70 (eHsp70) has been shown to have protective or deleterious effects in cardiovascular events [15], [16], [36]–[39]. Some studies have shown that increased Hsp70 level was associated with low risk of cardiovascular diseases [15], [16], [36], [37]. In contrast, several studies including some from our lab suggested that eHsp70 might possibly play a harmful role in the pathogenesis and progression of cardiovascular diseases [38], [39]. Although autonomic dysfunction have been associated with and may even precede various forms of cardiovascular diseases [40], no study has reported the effect of plasma Hsp70 on HRV. In this study, we did not find significant association between Hsp70 and various HRV indices in total population. However, we observed significant and consistent interactions between multiple PAHs metabolites and Hsp70 on HRV. There was a dose-response increase in SDNN, LF and TP in relation to elevated Hsp70 levels when PAHs metabolites was low but not when it was high, suggesting that increased plasma Hsp70 may have played a protective role in autonomic function when workers were exposed to relatively low levels of PAHs. Our previous studies showed that exposure to coke oven emission resulted in a dose-dependent increase in levels of plasma Hsp70 [19], but we did not explore whether there was a reverse relationship between PAHs levels and Hsp70 when workers were exposed to low coke oven emission as the relatively small sample size (n = 101) [19]. We speculate that circulating Hsp70 levels can be in the range which has been shown to exert protective effect against light damage when exposure to low-PAHs levels, and subsequent increase of HRV.

Several limitations of the present study merit consideration. First, the cross-sectional design is not able to prove the existence of a causal relationship whether inflammation leads to decreased HRV or vice versa. Second, monitoring of the 5-min HRV represents only short-term cardiac autonomic nerve modulation and the definitive clinical significance should be elucidated. Third, we did not adjust for multiple testing because of a high correlation both in various HRV indices and in PAHs metabolites. Over adjustment for multiple comparisons may increase the type II error and render truly important associations insignificant. Lastly, we could not rule out residual and unmeasured confounders, although we adjusted for a wide range of confounding factors.

## Conclusions

In this study, we observed the dose-response relationships between urinary OH-PAHs and IL-6 as well as IL-6 and HRV indices. In particular, a prominent effect of IL-6 on HRV was found in the high-PAHs metabolites groups, whereas high Hsp70 levels may have a protective effect on HRV in low-PAHs metabolites groups. However, further researches are needed to confirm these findings in prospective studies and elucidate the possible mechanisms of these associations.

## Supporting Information

File S1 **This file contains Table S1 through Table S3.** Table S1, Urinary levels of PAHs metabolites at different working sites. Table S2, The correlation coefficients (r) among individual PAHs metabolites and ΣOH-PAHs. Table S3, Effects of PAHs metabolites on HRV.
(DOC)
