# Supplementary material for: Association of Plasma IL-6 and Hsp70 with HRV at Different Levels of PAHs Metabolites
Source: PLoS One. 2014 Apr 10;9(4):e92964. doi: 10.1371/journal.pone.0092964 (PMC3982957; doi:10.1371/journal.pone.0092964)
Supplement: File S1 — This file contains Table S1 through Table S3. Table S1, Urinary levels of PAHs metabolites at different working sites. Table S2, The correlation coefficients (r) among individual PAHs metabolites and ΣOH-PAHs. Table S3, Effects of PAHs metabolites on HRV. (DOC) [file pone.0092964.s001.doc]

**Table S1.** Urinary levels of PAHs metabolites at different working sites*

| PAHs metabolites | Control group (n = 272) | Exposure groups | | | *P*_trend_^a^ |
| --- | --- | --- | --- | --- | --- |
| (μg/mmol creatinine) |  | Low (n = 328) | Intermediate (n = 163) | High (n = 37) |  |
| 1-OHNa | 1.32 (0.80, 2.16) | 1.48 (0.86, 2.72) | 1.94 (1.32, 3.37) | 2.41 (1.76, 4.28) | **< 0.001** |
| 2-OHNa | 1.38 (0.70, 2.15) | 1.36 (0.73, 2.29) | 2.12 (1.22, 3.29) | 3.13 (1.73, 4.94) | **< 0.001** |
| 2-OHFlu | 0.72 (0.50, 1.06) | 0.88 (0.60, 1.41) | 1.02 (0.65, 1.62) | 1.36 (0.93, 2.19) | **< 0.001** |
| 9-OHFlu | 0.45 (0.12, 1.02) | 0.56 (0.25, 1.41) | 0.57 (0.25, 1.19) | 0.71 (0.34, 1.08) | **0.006** |
| 1-OHPh | 0.81 (0.33, 1.48) | 0.74 (0.32, 1.36) | 0.99 (0.61, 1.96) | 1.47 (0.80, 2.59) | **< 0.001** |
| 2-OHPh | 0.25 (0.16, 0.48) | 0.30 (0.19, 0.50) | 0.35 (0.22, 0.70) | 0.52 (0.33, 0.76) | **< 0.001** |
| 3-OHPh | 0.30 (0.19, 0.47) | 0.35 (0.17, 0.60) | 0.43 (0.22, 0.83) | 0.71 (0.40, 1.21) | **< 0.001** |
| 4-OHPh | 0.36 (0.12, 0.77) | 0.34 (0.13, 0.70) | 0.26 (0.12, 0.60) | 0.38 (0.28, 0.61) | 0.642 |
| 9-OHPh | 0.64 (0.36, 1.27) | 0.68 (0.37, 1.23) | 0.85 (0.42, 1.47) | 0.96 (0.60, 1.68) | **0.007** |
| 1-OHP | 2.81 (1.62, 6.11) | 3.06 (1.80, 5.24) | 3.75 (2.22, 6.91) | 4.97 (3.44, 7.63) | **< 0.001** |
| ΣOH-PAHs | 10.41 (6.90, 17.59) | 11.07 (7.44, 17.42) | 13.46 (8.93, 21.52) | 16.78 (12.47, 27.95) | **< 0.001** |

^*^Office workers was defined as the control group, and workers at adjunct-oven, and at side and bottom-oven, and top-oven were defined as low, intermediate and high exposure groups, respectively. Values are presented as median (25th, 75th quartile).

^a^Simple linear regression for the trend of urinary PAHs metabolites with the exposure levels.

**Table S2.** The correlation coefficients (r) among individual PAHs metabolites and ΣOH-PAHsa

|  | 1-OHNa | 2-OHNa | 2-OHFlu | 9-OHFlu | 1-OHPh | 2-OHPh | 3-OHPh | 4-OHPh | 9-OHPh | 1-OHP | ΣOH-PAHs |
| --- | --- | --- | --- | --- | --- | --- | --- | --- | --- | --- | --- |
| 1-OHNa | 1.000 | 0.821^**^ | 0.518^**^ | 0.298^**^ | 0.562^**^ | 0.612^**^ | 0.457^**^ | 0.156^**^ | 0.595^**^ | 0.694^**^ | 0.851^**^ |
| 2-OHNa |  | 1.000 | 0.470^**^ | 0.231^**^ | 0.524^**^ | 0.471^**^ | 0.360^**^ | 0.107^*^ | 0.459^**^ | 0.585^**^ | 0.772^**^ |
| 2-OHFlu |  |  | 1.000 | 0.342^**^ | 0.296^**^ | 0.561^**^ | 0.513^**^ | 0.074^*^ | 0.454^**^ | 0.410^**^ | 0.601^**^ |
| 9-OHFlu |  |  |  | 1.000 | 0.253^**^ | 0.174^**^ | **-0.022** | 0.305^**^ | 0.303^**^ | 0.229^**^ | 0.477^**^ |
| 1-OHPh |  |  |  |  | 1.000 | 0.441^**^ | 0.165^**^ | 0.108^*^ | 0.542^**^ | 0.629^**^ | 0.702^**^ |
| 2-OHPh |  |  |  |  |  | 1.000 | 0.644^**^ | **0.060** | 0.660^**^ | 0.618^**^ | 0.651^**^ |
| 3-OHPh |  |  |  |  |  |  | 1.000 | **0.023** | 0.407^**^ | 0.367^**^ | 0.418^**^ |
| 4-OHPh |  |  |  |  |  |  |  | 1.000 | 0.122^**^ | 0.152^**^ | 0.262^**^ |
| 9-OHPh |  |  |  |  |  |  |  |  | 1.000 | 0.645^**^ | 0.700^**^ |
| 1-OHP |  |  |  |  |  |  |  |  |  | 1.000 | 0.836^**^ |
| ΣOH-PAHs |  |  |  |  |  |  |  |  |  |  | 1.000 |

^*^*P* < 0.05 and ^**^*P* < 0.001.

^a^Pearson partial correlation, adjusting for age, sex, length of work, smoking status, alcohol use, BMI, physical activity, working sites, workshift and weekday.

**Table S3.** Effects of PAHs metabolites on HRV*

| HRV indices | Quartiles of PAHs metabolites | | | | *P*_trend_^a^ |
| --- | --- | --- | --- | --- | --- |
|  | Q1 | Q2 | Q3 | Q4 |  |
| 2-OHNa |  |  |  |  |  |
| SDNN | 3.69 (0.03) | 3.70 (0.03) | 3.64 (0.03) | 3.66 (0.03) | 0.259 |
| RMSSD | 3.19 (0.04) | 3.18 (0.03) | 3.13 (0.03) | 3.15 (0.03) | 0.305 |
| TP | 6.96 (0.08) | 6.97 (0.07) | 6.88 (0.07) | 6.91 (0.06) | 0.472 |
| LF | 5.80 (0.09) | 5.81 (0.08) | 5.65 (0.08) | 5.68 (0.08) | 0.138 |
| HF | 4.99 (0.10) | 4.95 (0.09) | 4.78 (0.09) | 4.77 (0.08) | **0.028** |
| 1-OHPh |  |  |  |  |  |
| SDNN | 3.67 (0.03) | 3.66 (0.03) | 3.67 (0.03) | 3.67 (0.03) | 0.860 |
| RMSSD | 3.19 (0.03) | 3.14 (0.03) | 3.16 (0.03) | 3.16 (0.03) | 0.745 |
| TP | 6.94 (0.07) | 6.95 (0.07) | 6.92 (0.07) | 6.90 (0.06) | 0.552 |
| LF | 5.74 (0.09) | 5.73 (0.08) | 5.73 (0.08) | 5.70 (0.08) | 0.582 |
| HF | 5.04 (0.09) | 4.88 (0.09) | 4.83 (0.09) | 4.77 (0.08) | **0.012** |

^*^HRV indices were natural log transformed and values are presented as multivariate adjusted means and SE.

^a^General linear models with adjustment for age, sex, length of work, smoking status, alcohol use, BMI, physical activity, working sites, workshift and weekday.
